# Enzymatic Methoxycarbonylation of Tyrosol and Hydroxytyrosol

**DOI:** 10.3390/ijms251810057

**Published:** 2024-09-19

**Authors:** Lucia Černáková, Michaela Macková, Tatiana Klempová, Peter Haluz, Vladimír Mastihuba, Mária Mastihubová

**Affiliations:** 1 Institute of Chemistry, Slovak Academy of Sciences, Dúbravská Cesta 9, 845 38 Bratislava, Slovakia; lucia.cernakova@savba.sk (L.Č.); peter.haluz@savba.sk (P.H.); vladimir.mastihuba@savba.sk (V.M.); 2 Institute of Biotechnology, Faculty of Chemical and Food Technology, Slovak University of Technology, Radlinského 9, 812 37 Bratislava, Slovakia; xmackova@gmail.com (M.M.); tatiana.klempova@stuba.sk (T.K.)

**Keywords:** lipase, tyrosol, hydroxytyrosol, dimethyl carbonate, methoxycarbonylation

## Abstract

Tyrosol and hydroxytyrosol are powerful phenolic antioxidants occurring in olive oil and in by-products from olive processing. Due to their high polarity, esterification or other lipophilization is necessary to make them compatible with lipid matrices. Hydroxytyrosol methyl carbonate is a more effective antioxidant than dibutylhydroxytoluene or α-tocopherol and together with tyrosol methyl carbonate exerts interesting pharmacological properties. The purpose of this work was the enzymatic preparation of alkyl carbonates of tyrosol and hydroxytyrosol. A set of 17 hydrolases was tested in the catalysis of tyrosol methoxycarbonylation in neat dimethyl carbonate to find an economically feasible alternative to the recently reported synthesis of methyl carbonates catalyzed by Novozym 435. Novozym 435 was, however, found to be the best performing catalyst, while Novozym 735, pig pancreatic lipase, lipase F-AK and Lipex 100T exhibited limited reactivity. No enzyme accepted 1,2-propylene carbonate as the acylation donor. Under optimized reaction conditions, Novozym 435 was used in the batch preparation of tyrosol methyl carbonate and hydroxytyrosol methyl carbonate in quantitative yields. The enzymatic methoxycarbonylation of tyrosol and hydroxytyrosol can also be used as a method for their selective protection in enzymatic syntheses of phenylethanoid glycosides catalyzed with enzymes comprising high levels of acetyl esterase side activity.

## 1. Introduction

**Figure 1:**
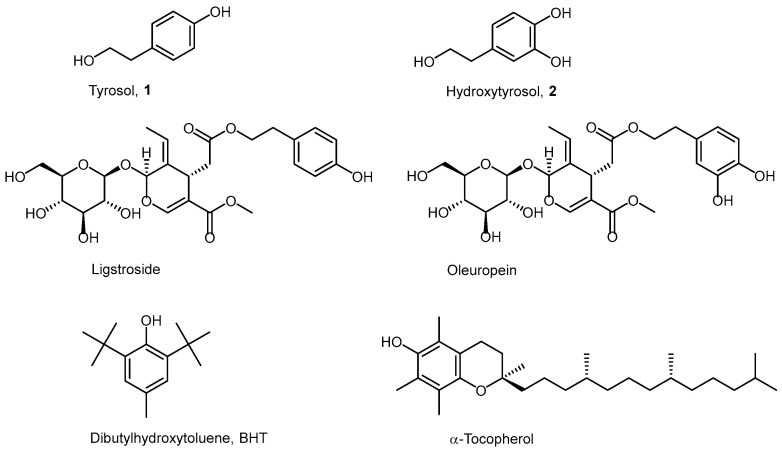
Natural and synthetic antioxidants tyrosol (**1**), hydroxytyrosol (**2**), ligstroside, oleuropein, BHT and α-tocopherol.

Tyrosol, 2-(4-hydroxyphenyl)ethanol (**1**), and hydroxytyrosol, 2-(3,4-dihydroxyphenyl)ethanol (**2**), are phenolic alcohols widely abundant as aglycones in phenylethanoid glycosides (PEGs), biologically active secondary metabolites of medicinal plants [1,2,3]. They also occur as antioxidant substances in olive trees and olive products, where they appear free or esterified in the form of olive secoiridioids oleuropein and ligstroside (Figure 1) [4,5]. Hydroxytyrosol is considered one of the strongest antioxidants in nature (as potent an antioxidant as dibutylhydroxytoluene, BHT), with strong antiproliferative and anti-inflammatory activities [4]. On the other hand, tyrosol, despite being a significantly weaker antioxidant, exerts a powerful protective effect against oxidative injuries in cell systems [6] and improves intracellular antioxidant defense systems [7].

In connection with constraints on the toxicity and allergenicity of synthetic antioxidants used in the cosmetic industry [8,9], natural substances and their derivatives are in the spotlight as alternatives. Despite having great potential for applications in the food and cosmetic industries, due to their high polarities, hydroxytyrosol and tyrosol have poor compatibility with lipids or cosmetic emulsions. Modifications such as acylation [10,11,12], alkylation [13] or arylation [14] provide effective lipid antioxidants, but the chemical procedures used may barely be considered eco-friendly when using catalysts and reagents such as DBU, i.e., 1,8-diazabicyclo(5.4.0)undec-7-ene [10], anhydrides in pyridine [12], arylhalogenides or alkylhalogenides combined with strong bases [13], or palladium complexes in dioxane [14]. Therefore, enzymatic acylations by various aliphatic and unsaturated acids or oil-derived mixtures were proposed as green and biocompatible methods of modification of **1** and **2** [10,15,16,17]. Although the acylation of hydroxytyrosol significantly increased its antioxidant effect toward lipids, the acylation of tyrosol has the opposite effect, in both cases independently of the chain length or chemical nature of the acyl substituent [10]. Alkyl carbonates of **2** represent an interesting group of acylated phenylethanoids with a higher antioxidant effect than that of BHT or α-tocopherol [12]. Methyl carbonates of **1** and **2** exert a significant antiproliferative effect in different tumor cell lines *in vitro*, considerably higher compared to free **1** and **2** [18].

Very recently, Vicinanza et al. [19] reported the flow synthesis of methyl carbonates and symmetric carbonates of **1** and **2** catalyzed by lipase B from *Candida antarctica* (Novozym 435) in neat dimethyl carbonate (DMC). The ability of ester hydrolases to use carbonate esters as acyl donors was reported for the first time by Abramowicz [20]. Since then, several enzymes have been reported to catalyze the alkyloxycarbonylation and aryloxycarbonylation of alcohols, including lipases from *Aspergillus niger* [21,22], *Thermomyces lanuginosus* [23], *Pseudomonas cepacia* and *Candida antarctica* lipases A and B [24]. Apparently, the ability to use organic carbonates as substrates for transesterification is not universal among lipases and esterases.

In our attempts to enzymatically prepare various tyrosol and hydroxytyrosol glycosides as building blocks for bioactive PEGs, we have found two unique biocatalysts capable of catalyzing glycosylation of phenolic hydroxyl of tyrosol—a commercial mixture of glycosidases Aromase H2 and seeds of common buckthorn (*Rhamnus cathartica*) [25,26]. The transglycosylations catalyzed by these catalysts were, however, non-selective, providing mixtures of phenyl and alkyl acuminosides, robinobiosides and rutinosides of tyrosol. Since Aromase H2 also comprises high levels of acetyl esterase, selective acetylation of either phenol or primary hydroxyl of tyrosol does not provide reliable protection to obtain a sole glycoside. Enzymatic methoxycarbonylation in neat dimethyl carbonate may therefore be a useful green method of selective protection of primary hydroxyls, avoiding other ester hydrolases to hydrolyze the methoxycarbonyl protecting group. The flow method of tyrosol and hydroxytyrosol methoxycarbonylation reported by Vicinanza et al. [19] is more appropriate for large-scale processes. The continual process on a small scale requires a sensitively controlled feeding system; on a larger scale, it requires rather high amounts of reactants until the equilibrium is reached. The great advantage of the flow process is the reduced amount of immobilized catalyst necessary to execute the reaction. This advantage may be compensated in the batch system, if the enzyme is recyclable by simple filtering and washing. The purpose of this paper is therefore to optimize batch enzymatic preparation of selectively methoxycarbonylated tyrosol and hydroxytyrosol and to search for a less expensive catalyst for this process.

## 2. Results and Discussion

To test the ability of lipases to accept alkyl carbonates as substrates for acylations (alkoxycarbonylations) of tyrosol, 17 lipases and esterase-comprising enzyme mixes were tested in the acylation of **1** in DMC and 1,2-propylene carbonate. TLC was used to evaluate the first screening. The selection of enzymes was guided by an effort to cover ester hydrolases of fungal, bacterial, yeast and animal origin as a more economical alternative to Novozym 435 used by Vicinanza et al. [19], preferably ones accepting short fatty acids or primary hydroxyls of the alcohol (sn-1,3specificity on triglycerides). Lipases reported to catalyze acylations by DMC or other carbonate esters [20,21,22,23,24] were included in the screening as well (Table 1).

**Figure 2:**
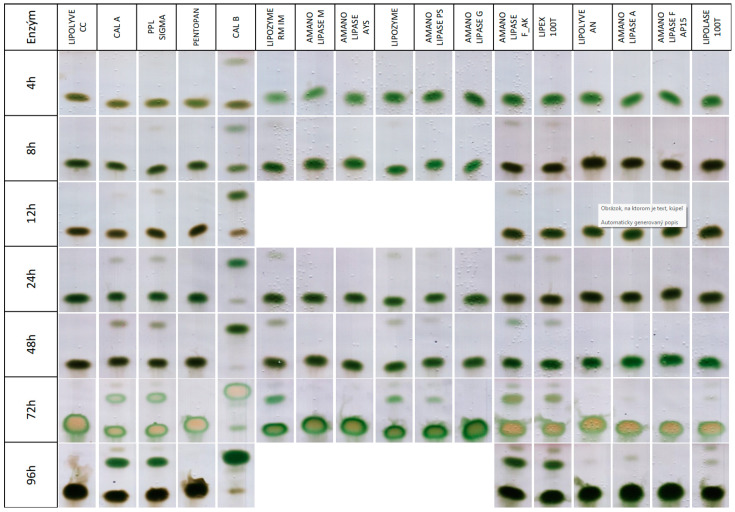
TLC screening of lipase-catalyzed methoxycarbonylations of tyrosol.

While no tyrosol acylation occurred in 1,2-propylene carbonate, several lipases catalyzed the methoxycarbonylation of **1** in DMC (Figure 2). The lipase B from *Candida antarctica* (Novozym 435) showed the best performance, while significantly lower methoxycabonylating activity was observed for Novozym 735 (lipase A from *Candida antarctica*), pig pancreatic lipase, lipase F-AK and Lipex 100T (Figure 2). Contrary to observations by Tudorache et al. [21,22] who successfully used lipase from *Aspergillus niger* in the methoxycarbonylation of glycerol, neither lipase A nor Lipolyve AN (both from *A. niger*) catalyzed the acylation of tyrosol. Similarly, neither of the inexpensive enzymes from *Thermomyces lanuginosus* catalyzed the reaction, although Lee et al. [24] report the reactivity of Lipex 100 L from the same strain with DMC.

**Figure 3:**
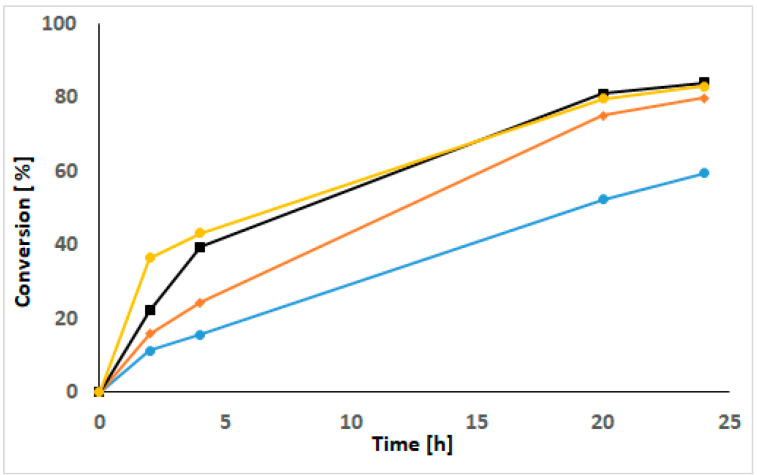
Time course of methoxycarbonylation of tyrosol catalyzed by Novozym 435—effect of enzyme load. Concentration of tyrosol in DMC: 10 mg/mL. Load of Novozym 435: 5 mg/mL (-●-), 10 mg/mL (-♦-), 15 mg/mL (-■-) and 20 mg/mL (-●-).

These results confirm the validity of the choice of Novozym 435 as a catalyst for the continual methoxycarbonylation of tyrosol by Vicinanza et al. [19]. Despite its price, Novozym 435 in dimethyl carbonate was therefore selected as a catalyst for our further experiments with batch acylation of tyrosol. The standard of tyrosol methyl carbonate obtained in a nonoptimized reaction was used to select the reaction conditions. Dimethyl carbonate served both as a reaction medium and as an acyl (i.e., methoxycarbonyl) donor. Four loads of the enzyme were tested in the methoxycarbonylation of tyrosol at a concentration of 10 mg/mL. All enzyme concentrations resulted in conversions higher than 60% after 24 h (Figure 3). Within another 24 h, all reactions reached completion, with no free tyrosol remaining.

**Figure 4:**
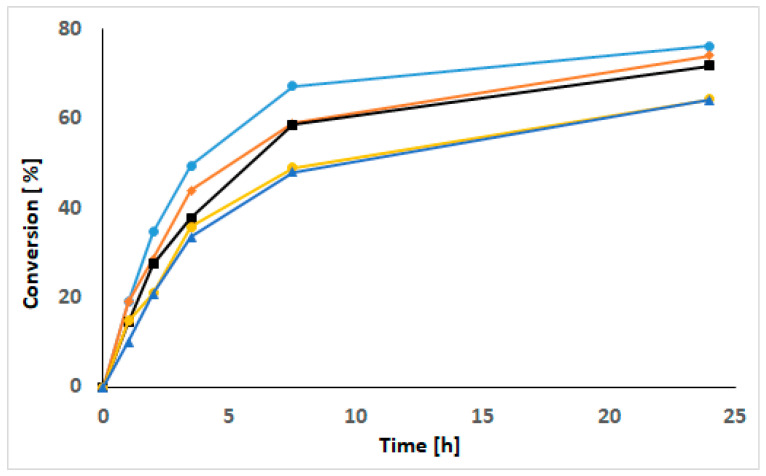
Time course of methoxycarbonylation of tyrosol catalyzed by Novozym 435—effect of tyrosol concentration. Load of Novozym 435: 20 mg/mL. Concentration of tyrosol in DMC: 5 mg/mL (-●-), 10 mg/mL (-♦-), 15 mg/mL (-■-), 20 mg/mL (-●-) and 25 mg/mL (-▲-).

**Scheme 1:**
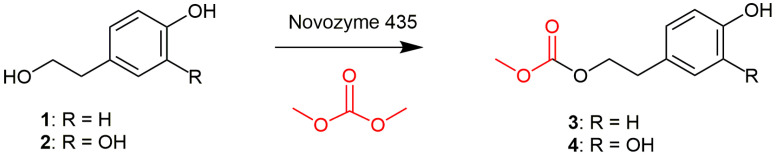
Methoxycarbonylation of **1** and **2** catalyzed by Novozym 435.

The speed of methoxycarbonylation of **1** decreased in proportion to the increase in its concentration (5 to 25 mg/mL; enzyme load 20 mg/mL), reaching conversion above 64% within 24 h in all experiments (Figure 4). The preparative reaction was realized at the 20 mL scale, with a tyrosol concentration of 25 mg/mL and an enzyme load of 20 mg/mL. The reaction was faster compared to the optimization experiments, probably due to better mixing in the stirred 20 mL batch; within 18 h, after isolation, it gave almost a quantitative yield of tyrosol methyl carbonate. The reaction provided only product **3** methoxycarbonylated on the primary hydroxyl of tyrosol. No product with acylated phenolic hydroxyl was found in the reaction mixture by HPLC analysis. The enzyme was therefore selective toward the acylation of primary hydroxyl and used only dimethyl carbonate as the acylation reagent (Scheme 1). The formation of dityrosol carbonate was not observed. This fact is in agreement with observations made by Vicinanza et al. [19], who obtained dityrosol carbonate only after a secondary reaction of **3** with tyrosol in tertiary butanol.

**Figure 5:**
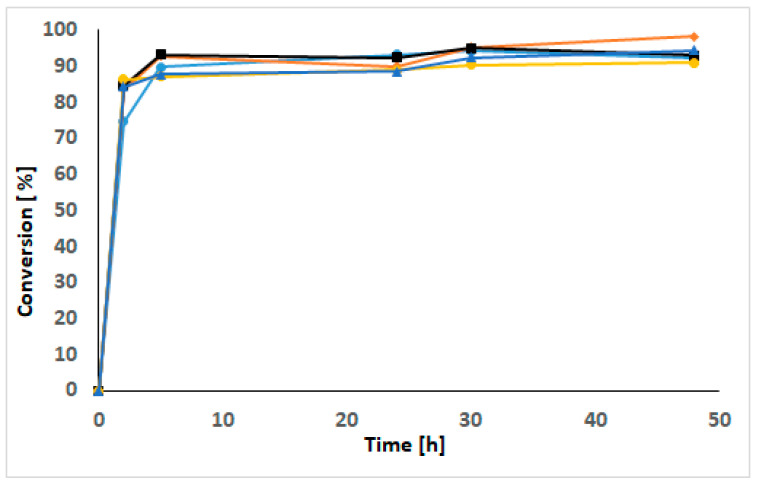
Time course of methoxycarbonylation of hydroxytyrosol catalyzed by Novozym 435—effect of enzyme load. Concentration of hydroxytyrosol in DMC: 10 mg/mL. Load of Novozym 435: 10 mg/mL (-●-), 20 mg/mL (-♦-), 30 mg/mL (-■-), 40 mg/mL (-●-) and 50 mg/mL (-▲-).

**Figure 6:**
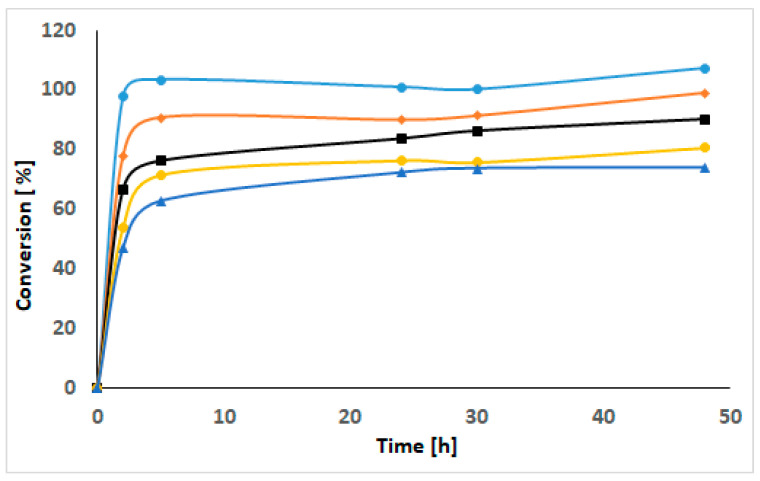
Time course of methoxycarbonylation of tyrosol catalyzed by Novozym 435—effect of tyrosol concentration. Load of Novozym 435: 20 mg/mL. Concentration of tyrosol in DMC: 5 mg/mL (-●-), 10 mg/mL (-♦-), 15 mg/mL (-■-), 20 mg/mL (-●-) and 25 mg/mL (-▲-).

The course of methoxycarbonylation of hydroxytyrosol **2** was more dependent on its starting concentration, probably due to the substrate inhibition of the enzyme. When the starting concentration of **2** in the reaction mixture was as low as 10 mg/mL, the reaction course was almost independent of the enzyme load (Figure 5), reaching within 5 h conversion above 80% and significantly slowing down, going almost to completion within 48 h of the overall reaction time. When the starting concentration of **2** was elevated to 25 mg/mL, the conversion did not exceed 75% (Figure 6). Therefore, a preparative reaction was carried out at a low starting concentration of **2** and with a high load of the enzyme, giving almost a quantitative yield of hydroxytyrosol methyl carbonate **4**. Again, **4** was the only reaction product, and no acylation of phenolic hydroxyl nor the formation of dihydroxytyrosol carbonate occurred (Scheme 1).

Our results confirm some exceptionality of Novozym 435 and generally of lipase B from *Candida antarctica*. This enzyme, possessing wide substrate specificity and on the other hand narrow regio- and stereospecificity, has plenty of applications from laboratory to industrial [27,28,29]. Although other enzymes were also reported to catalyze the acylation of various substrates with alkyl carbonates, Novozym 435 was thus found to be by far the best catalyst for the methoxycarbonylation of **1** and **2**. With such excellent performance in acylation with DMC, it is a prospective catalyst for industrial alkoxycarbonylations of diols and triols to produce polymeric carbonates [30]. On the laboratory level, the enzyme can be used for selective hydroxyl protection when standard acetylation is not appropriate.

As an example, in our attempts to hydrolyze hydroxytyrosol methyl carbonate **3** by Aromase H2 and by buckthorn seed meal, the carbonate remained intact and no hydrolysis occurred within 24 h. Since these two tested materials comprise interesting diglycosidase activities able to glycosylate the phenolic moiety of tyrosol [25,26], the enzymatic methoxycarbonylation of **1** and **2** can be used for the protection of its primary hydroxyl in enzymatic glycosylations of their phenolic moieties without being affected by the high level of acetyl esterase in Aromase H2.

## 3. Materials and Methods

### 3.1. Apparatus

High-performance liquid chromatography was performed on an Agilent 1200 Series apparatus (Agilent Technologies, Inc., Santa Clara, CA, USA) consisting of a quaternary pump, RI and UV–vis detectors, a column thermostat and a Rheodyne injector with a 20 µL loop. Flash chromatography was performed on an Isolera One from Biotage (Uppsala, Sweden), with UV detection at 265 nm using 25 g KP-Sil SNAP cartridges. The structures of the products were determined by a combination of ^1^H and ^13^C NMR spectroscopy as well as two-dimensional homonuclear and heteronuclear techniques (COSY, HSQC), and recorded on a 400 MHz Bruker AVANCE III HD equipped with a Prodigy CryoProbe (both from Bruker GmbH, Karlsruhe, Germany).

### 3.2. Enzymes and Chemicals

Lipase A (*Aspergillus niger*), Lipase F-AK (*Pseudomonas fluorescens*), Lipase AYS (*Candida rugosa*), Lipase F-AP15 (*Rhizopus oryzae*), Lipase G (*Penicillium camemberti*), Lipase M (*Mucor javanicus*) and Lipase PS (*Burkholderia/Pseudomonas cepacia*) were from Amano (Elgin, IL, USA), lipases Lipolyve AN (*Aspergillus niger*) and Lipolyve CC (*Candida cylindracea*) were from Lyven (Colombelles, France), lipases Lipex 100T, Lipolase 100T and Lipozyme TL-IM (all *Thermomyces lanuginosus*), Novozym 735, Novozym 435 (both *Candida antarctica*), Lipozyme RM-IM (*Rhizomucor miehei*) and xylanase Pentopan 500 BG (*Thermomyces lanuginosus*) were from Novozymes (Bagsvaerd, Denmark), and pig pancreatic lipase was from Sigma Aldrich (St. Louis, MO, USA).

Tyrosol (97%) was purchased from Maybridge (Loughborough, Leicestershire, UK), DMC and 1,2-propylene carbonate were from Merck KGaA (Darmstadt, Germany). Silica gel 60 for flash chromatography was obtained from Fluka (Buchs, Switzerland). TLC was performed on alumina plates with Silica gel 60 F254 from Merck KGaA (Darmstadt, Germany).

### 3.3. Synthesis of Hydroxytyrosol and Standards of Tyrosol Methyl Carbonate and Hydroxytyrosol Methyl Carbonate

Hydroxytyrosol was prepared according to Zhang et al. (2010) [31]; standards of tyrosol methyl carbonate and hydroxytyrosol methyl carbonate were prepared by nonoptimized enzymatic reactions catalyzed by Novozym 435.

### 3.4. Enzymatic Reactions

#### 3.4.1. Screening of Enzymes for Methoxycabonylations of Tyrosol

Portions of DMC or 1,2-propylene carbonate (350 µL) comprising 5 mg of tyrosol were added to 5 mg of a tested enzyme. The reaction mixtures were incubated at 37 °C and 500 rpm in a thermoshaker. At predetermined time intervals, 5 µL of the reaction mixture was withdrawn, quenched by mixing with 200 µL of methanol and spotted on alumina plates with Silica gel 60 F254 from Merck KGaA (Darmstadt, Germany). The plates were eluted with a mixture of chloroform and methanol (6:1), dried, dipped into the staining solution (5.16 g MnCl_2_. 4 H_2_O; 500 mL methanol; 33 mL sulphuric acid; 465 mL distilled water) and heated at ca. 130 °C.

#### 3.4.2. Screening of Reaction Conditions for Methoxycabonylations of Tyrosol and Hydroxytyrosol

Reaction mixtures in 1.5 mL Eppendorf tubes comprised 1 mL DMC and predefined amounts of Novozym 435 and tyrosol or hydroxytyrosol. The reaction mixtures were shaken at 37 °C and 500 rpm. At predefined time intervals, 50 µL portions of the reaction mixtures were withdrawn, mixed with 550 µL acetonitrile and passed through 0.22 µm syringe filters. A total of 300 µL of filtrates was mixed with 700 µL of distilled water. The processed reaction mixtures were analyzed by HPLC on a NUCLEOSIL^®^ 100–5 C8 (5 μm) column (Merck KGaA, Darmstadt, Germany) equilibrated to 35 °C and eluted with 30% acetonitrile in water at a flow rate of 1 mL/min. Tyrosol, hydroxytyrosol and their methyl carbonates were detected at 265 nm and quantified from calibration curves of their pure standards. Each reaction was executed in two parallels.

#### 3.4.3. Preparation of Tyrosol Methyl Carbonate and Hydroxytyrosol Methyl Carbonate

Novozyme 435 (400 mg) was added to 500 mg of tyrosol dissolved in 20 mL of DMC. The mixture was continually stirred at 37 °C and stopped after 18 h by filtering off the enzyme. The filter cake was washed with a small volume of chloroform, the washings were combined with the filtrate and organic solvents were removed by vacuum evaporation. Dry residue was redissolved in chloroform, and the product was separated by flash chromatography with a gradient of methanol in chloroform. Fractions containing the pure product were pooled and the organic solvents were evaporated to give 686 mg (97%) of pure tyrosol methyl carbonate **3**.

The reaction mixture for the methoxycarbonylation of hydroxytyrosol comprised 800 mg of Novozym 435 and 200 mg of hydroxytyrosol in 40 mL of DMC. After 18 h, the reaction was stopped by the same procedures as in the reaction with tyrosol, providing 263 mg (96%) of pure hydroxytyrosol methyl carbonate **4**.

#### 3.4.4. Enzymatic Hydrolysis of Tyrosol Methyl Carbonate **3**

A total of 10 mg of **3** was dissolved in 0.5 mL of water in an Eppendorf tube and 10 mg of buckthorn seed meal was added. The reaction was shaken at 37 °C and the potential release of tyrosol was checked within 24 h by thin-layer chromatography. The hydrolysis of **3** by Aromase H2 was executed similarly.An acetate buffer of pH 5 (0.1 M) and a temperature of 50 °C was used. The reaction was started by adding 50 µL of Aromase H2 dissolved in water(10 mg/mL).

### 3.5. NMR Data of Products

#### 3.5.1. Tyrosol Methyl Carbonate (4-Hydroxyphenethyl Methyl Carbonate, **3**)

Amorphous white solid. ^1^H NMR (400 MHz, CDCl_3_), δ (ppm): 7.06 (d, *J* = 8.5 Hz, 2H, H-Ph), 6.77 (d, *J* = 8.5 Hz, 2H, H-Ph), 5.73 (s, 1H, OH), 4.30 (t, *J* = 7.1 Hz, 2H, CH_2_), 3.77 (s, 3H, CH_3_), 2.89 (t, *J* = 7.1 Hz, 2H, CH_2_). ^13^C NMR (101 MHz, CDCl_3_), δ (ppm): 155.9 (C=O), 154.5 (C-Ph), 130.0 (2xCH-Ph), 129.0 (C-Ph), 115.4 (2xCH-Ph), 68.8 (CH_2_), 54.8 (CH_2_), 34.2 (CH_3_). Spectra are in accordance with Vicinanza et al. [19]. For spectra recordings see Figures S1 and S2 in the Supplementary Materials.

#### 3.5.2. Hydroxytyrosol Methyl Carbonate (3,4-Dihydroxyphenethyl Methyl Carbonate, **4**)

Amorphous white solid. ^1^H NMR (400 MHz, (CD_3_)_2_CO), δ (ppm): 7.80 (s, 1H, OH), 7.77 (s, 1H, OH), 6.74 (d, *J* = 8.0 Hz, 1H, H-Ph), 6.74 (d, *J* = 2.1 Hz, 1H, H-Ph), 6.58 (dd, *J* = 8.1, 2.1 Hz, 1H), 4.23 (t, *J* = 7.1 Hz, 2H, CH_2_), 3.70 (s, 3H, CH_3_), 2.80 (t, *J* = 7.1 Hz, 2H, CH_2_). ^13^C NMR (101 MHz, (CD_3_)_2_CO) δ (ppm): 156.4 (C=O), 145.9 (C-Ph), 144.6 (C-Ph), 130.0 (C-Ph), 121.0 (CH-Ph), 116.8 (CH-Ph), 116.1 (CH-Ph), 69.3 (CH_2_), 54.8 (CH_3_), 35.1 (CH_2_). Spectra are in accordance with Vicinanza et al. [19]. For spectra recordings see Figures S3 and S4 in the Supplementary Materials.

## 4. Conclusions

From among 17 tested lipases and esterases, Novozym 435 was found to be the best biocatalyst for the methoxycarbonylation of tyrosol **1** and hydroxytyrosol **2**. The process proceeded selectively on the primary hydroxyls of acceptors **1** and **2,** and only their monosubstitution in dimethyl carbonate occurred. Although both reactions were fast and provided quantitative yields after optimization, the affinity of the catalyst to both substrates differed, being strongly dependent on substrate concentration in the case of methoxycarbonylation of hydroxytyrosol **2**. Interestingly, none of the tested enzymes reacted with 1,2-propylene carbonate. The simple enzymatic reactions presented in this work provide two products known for their strong antioxidant effects and pharmacological activities. Moreover, the selectivity of the reaction can be used for the protection of primary hydroxyls when acetylation is not appropriate. As an example, methoxycarbonylated products **3** and **4** may be used in the enzymatic preparation of PEGs selectively glycosylated on unprotected phenolic moieties, while the high level of acetyl esterase present in the used glycosidase does not affect the reaction.

## Figures and Tables

**Table 1:** Summary of lipases tested in methoxycarbonylations of tyrosol.

Lipase	Origin	Specificity/Application
Lipase A	*Aspergillus niger*	DMC [21,22]
Lipolyve AN	*Aspergillus niger*	DMC [21,22]
Lipase G	*Penicillium camemberti*	SCEs ^1^
Lipase F-AP15	*Rhizopus oryzae*	Sn-1,3
Lipozyme RM-IM	*Rhizomucor miehei*	SCEs ^1^
Lipase M	*Mucor javanicus*	Sn-1,3
Lipex 100T	*Thermomyces lanuginosus*	Sn-1,3
Lipolase 100T	*Thermomyces lanuginosus*	Sn-1,3
Lipozyme TL-IM	*Thermomyces lanuginosus*	Sn-1,3
Pentopan 500 BG	*Thermomyces lanuginosus*	Sn-1,3
Lipase AYS	*Candida rugosa (cylindracea)*	Diphenyl carbonate [20]
Lipolyve CC	*Candida rugosa (cylindracea)*	Diphenyl carbonate [20]
Novozym 735	*Candida antarctica* lipase A	Various carbonates [24]
Novozym 435	*Candida antarctica* lipase B	Various carbonates [19,23,24]
Lipase F-AK	*Pseudomonas fluorescens*	Non-specific, SCEs ^1^
Lipase PS	*Burkholderia cepacia*	DMC [24]
PPL	Pig pancreas	Sn-1,3

^1^ SCEs—short chain esters.

## Data Availability

Data are contained within the article.

## Supplementary Materials

The following supporting information can be downloaded at: https://www.mdpi.com/article/10.3390/ijms251810057/s1.
