# Supplementary material for: Enzymatic Methoxycarbonylation of Tyrosol and Hydroxytyrosol
Source: Int J Mol Sci. 2024 Sep 19;25(18):10057. doi: 10.3390/ijms251810057 (PMC11432353; doi:10.3390/ijms251810057)
Supplement: Supplementary file 1 [file ijms-25-10057-s001.zip › Supplementary Materials.pdf]

International Journal of Molecular Sciences

MDPI

Supplementary Materials

Enzymatic methoxycarbonylation of tyrosol and hydroxytyrosol

Lucia Černáková [1], Michaela Macková [2], Tatiana Klempová [2], Peter Haluz [1], Vladimír Mastihuba [1] and Mária Mastihubová [1,*]

[1] Institute of Chemistry, Slovak Academy of Sciences, Dúbravská cesta 9, SK-845 38 Bratislava, Slovakia
[2] Institute of Biotechnology, Faculty of Chemical and Food Technology, Slovak University of Technology, Radlinského 9, 812 37 Bratislava, Slovakia
* Correspondence: maria.mastihubova@savba.sk;

Table of Contents:

1. ^{1}H and ^{13}C NMR spectra of **3** S2

2. ^{1}H and ^{13}C NMR spectra of **4** S3

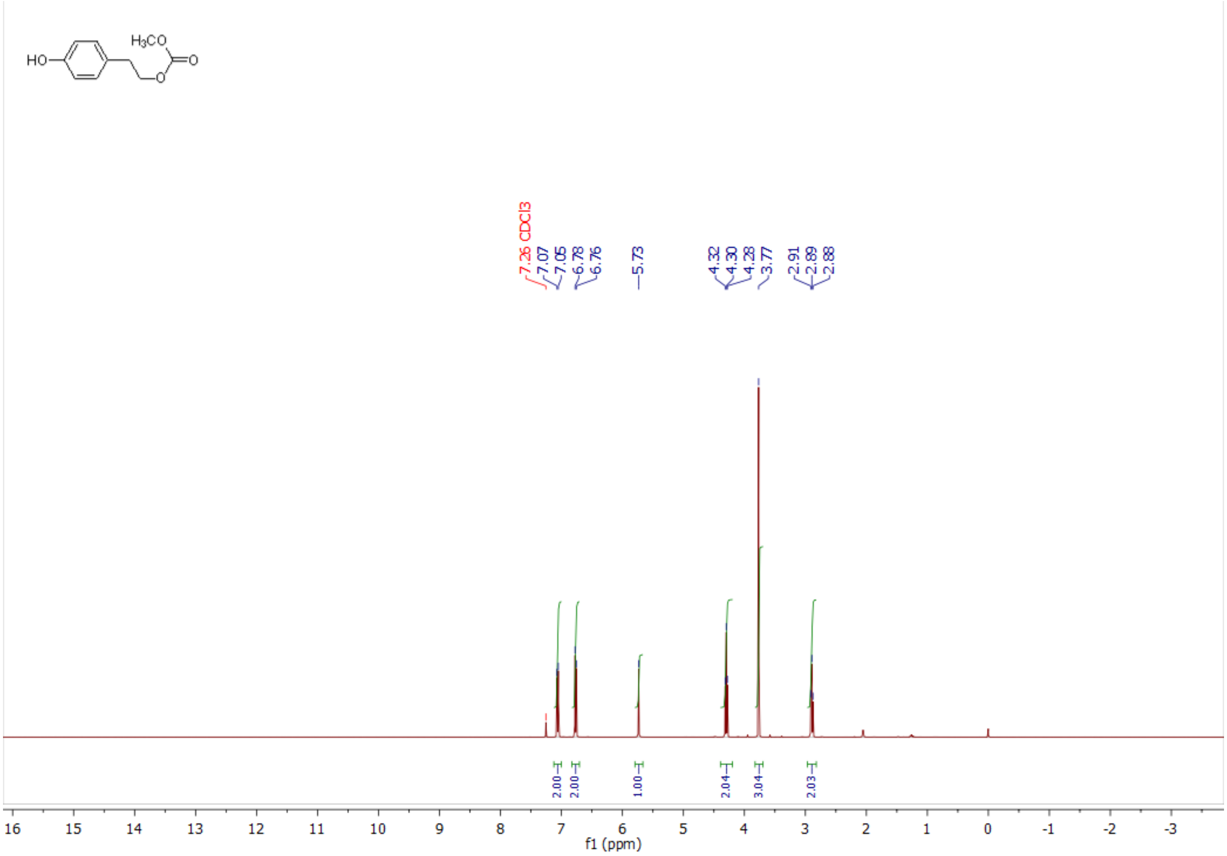

Figure S1. ^{1}H NMR of tyrosol methyl carbonate (**3**)

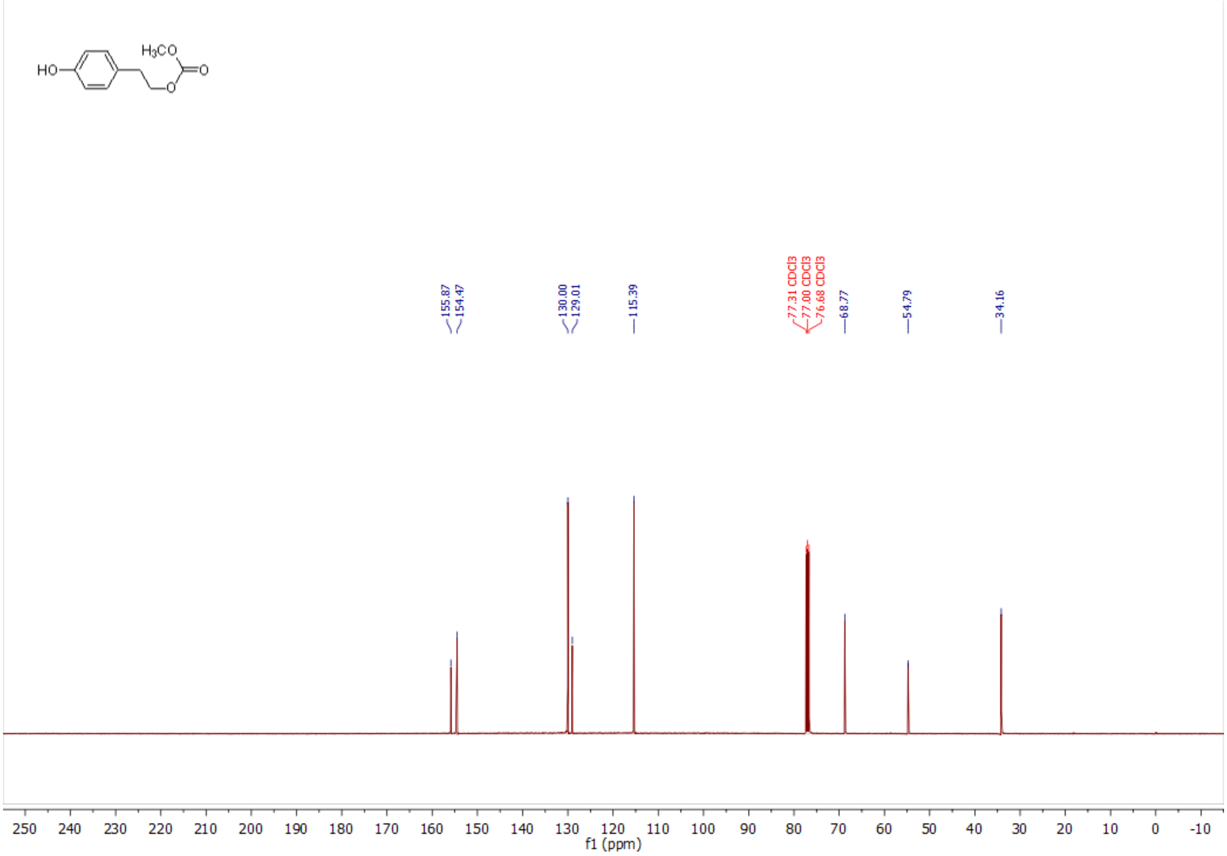

Figure S2. ^{13}C NMR of tyrosol methyl carbonate (**3**)

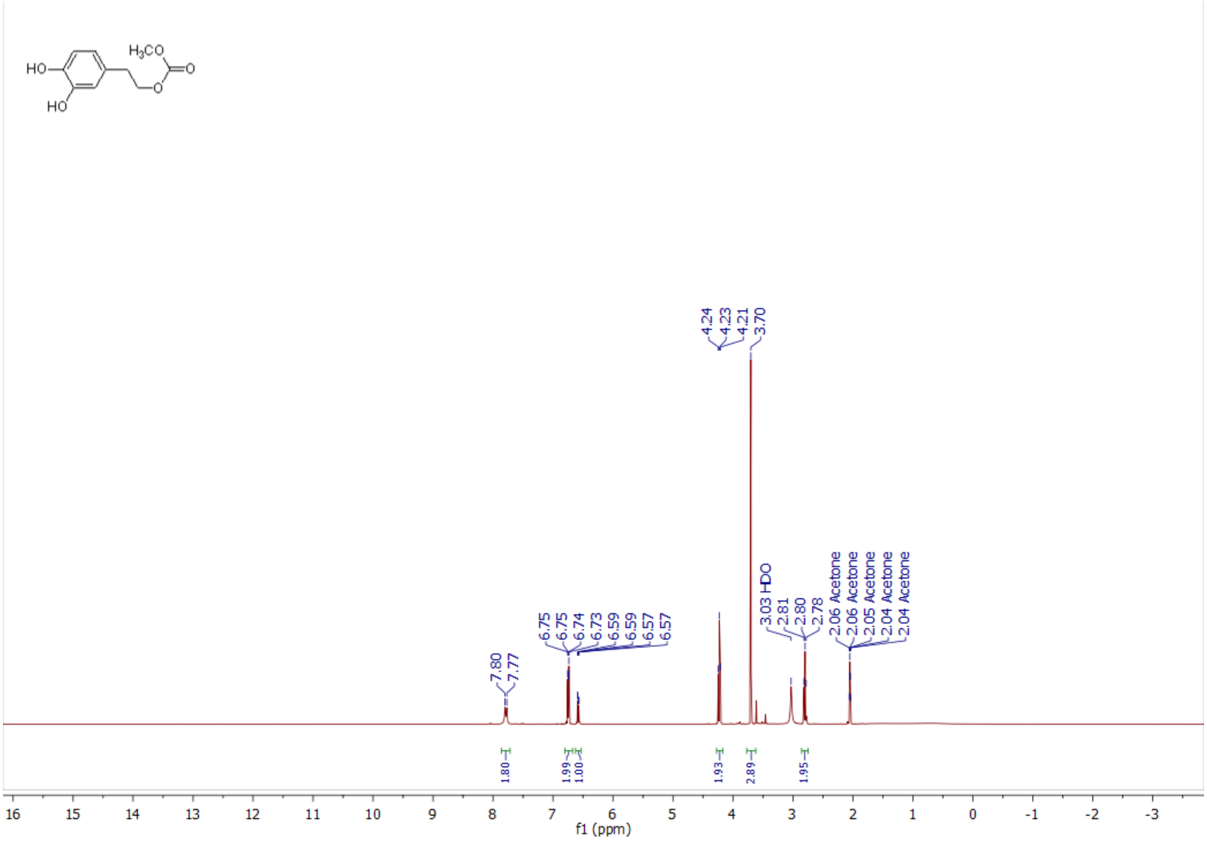

Figure S3. ^{1}H NMR of hydroxytyrosol methyl carbonate (**4**)

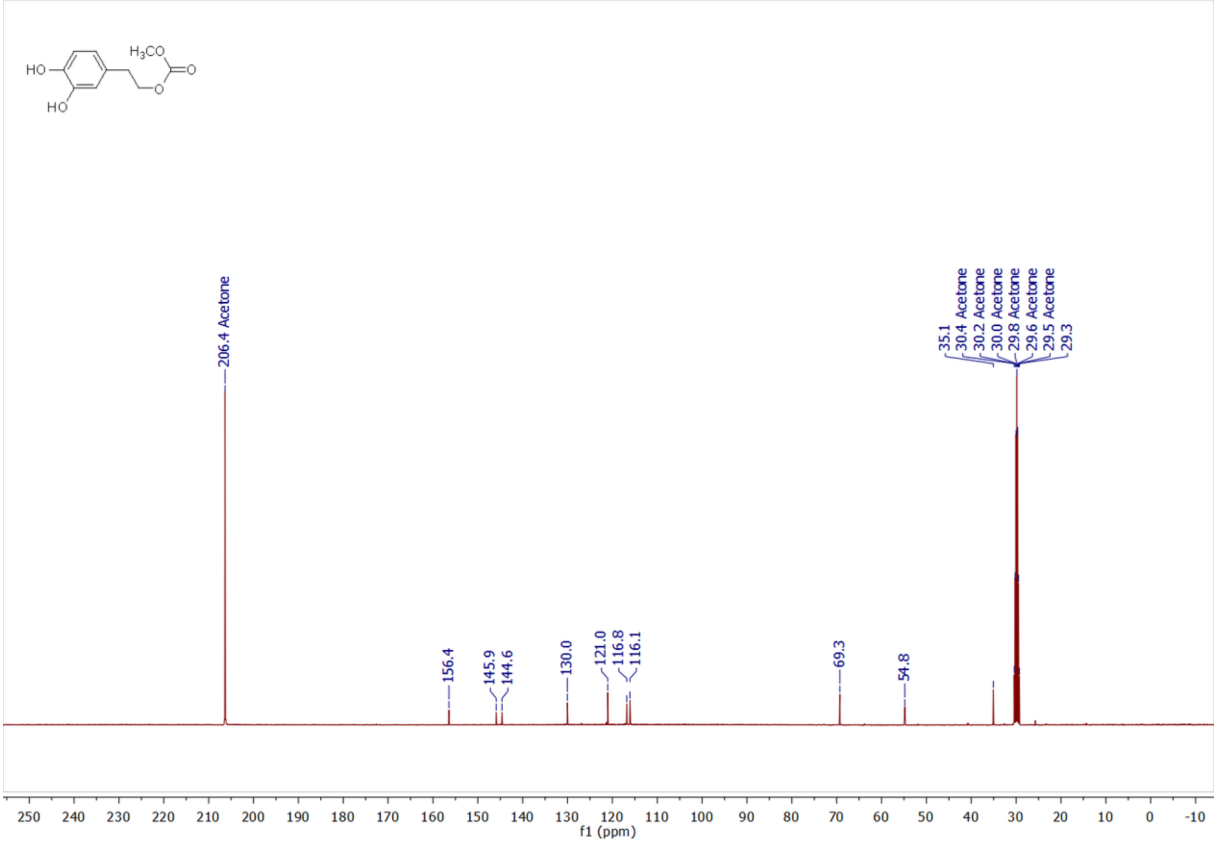

Figure S4. ^{13}C NMR of hydroxytyrosol methyl carbonate (**4**)
